# Design and Evaluation of Optimized Artificial HIV-1 Poly-T Cell-Epitope Immunogens

**DOI:** 10.1371/journal.pone.0116412

**Published:** 2015-03-18

**Authors:** Alena Reguzova, Denis Antonets, Larisa Karpenko, Alexander Ilyichev, Rinat Maksyutov, Sergei Bazhan

**Affiliations:** State Research Center of Virology and Biotechnology “Vector”, Koltsovo, Novosibirsk region, 630559, Russia; National Institute of Allergy and Infectious Diseases, UNITED STATES

## Abstract

A successful HIV vaccine in addition to induction of antibody responses should elicit effective T cell responses. Here we described possible strategies for rational design of T-cell vaccine capable to induce high levels of both CD4+ and CD8+ T- cell responses. We developed artificial HIV-1 polyepitope T-cell immunogens based on the conserved natural CD8+ and CD4+ T cell epitopes from different HIV-1 strains and restricted by the most frequent major human leukocyte antigen (HLA) alleles. Designed immunogens contain optimized core polyepitope sequence and additional “signal” sequences which increase epitope processing and presentation to CD8+ and CD4+ T-lymphocytes: N-terminal ubiquitin, N-terminal signal peptide and C-terminal tyrosine motif of LAMP-1 protein. As a result we engineered three T cell immunogens – TCI-N, TCI-N2, and TCI-N3, with different combinations of signal sequences. All designed immunogens were able to elicit HIV-specific CD4+ and CD8+ T cell responses following immunization. Attachment of either ubiquitin or ER-signal/LAMP-1 sequences increased both CD4+ and CD8+ mediated HIV-specific T cell responses in comparison with polyepitope immunogen without any additional signal sequences. Moreover, TCI-N3 polyepitope immunogen with ubiquitin generated highest magnitude of HIV-specific CD4+ and CD8+ T cell responses in our study. Obtained data suggests that attachment of signal sequences targeting polyepitope immunogens to either MHC class I or MHC class II presentation pathways may improve immunogenicity of T-cell vaccines. These results support the strategy of the rational T cell immunogen design and contribute to the development of effective HIV-1 vaccine.

## Introduction

Development of an effective immunoprophylactic vaccine against HIV-1 has been the major goal for researchers since the virus was discovered. Prior experience in the field of T cell-based vaccines has led to the conception that optimized HIV-1 immunogens should be able to elicit HIV-specific CD8+ cytotoxic T-lymphocyte (CTL) responses against wide range of HIV-1 strains. There is a general knowledge that CD8+ T cells are efficient mediators of antiviral immunity, and would therefore be an appropriate component of a T cell-based vaccine [1–3]. It was recently demonstrated that vaccine-mediated HIV-specific CD8+ T cell responses can control replication of HIV-1 in an animal model [4]. Furthermore, the rational vaccine design may improve its immunogenicity. Therefore, design of artificial polyepitope immunogens which induce CD8+ CTL responses against HIV-1 is a promising approach for the vaccine development [1].

There were a number of attempts to overcome antigenic variability of HIV-1 and to enhance immunogenicity of DNA-vaccines focused on CD8+ T cell responses [5–9]. First of all, taking into account high genetic variability of virus, to improve immunologic coverage an efficient T cell-based vaccine should contain conserved CD8+ T cell epitopes from different HIV-1 subtypes [10–12]. Secondly, optimized CD8+ T cell vaccines should be able to elicit CD8+ CTL responses against multiple epitopes from different HIV-1 proteins [11]. Furthermore, selected epitopes must be restricted by the most frequent major human leukocyte antigen (HLA) alleles to stimulate CD8+ T cell responses in the large-scale vaccination [13]. In addition, for polyepitope vaccine design it is crucial to include CD8+ CTL-epitopes with high binding affinity to major histocompatibility complex (MHC) class I molecules and capable to bind to several MHC allomorphs and to TAP (transporter associated with antigen processing) [14,15]. Besides, to enhance CD8+ T-lymphocyte responses immunogens should contain CD4+ T-helper epitopes restricted by MHC class II molecules. At the same time rational strategy to enhance vaccine immunogenicity may lead to high gene expression encoding target immunogens, as well as efficient processing of immunogenic proteins and presentation of released peptides (epitopes) in complex with MHC class I molecules to CD8+ T-lymphocytes [7,9,16–31]. Several approaches have to be used at the same time to improve immunogenicity of polyepitope candidate vaccines.

The aim of this research is to design polyepitope HIV-1 T cell immunogens with different strategies of their processing and presentation to CD8+ and CD4+ T-lymphocytes, to create DNA-vaccine constructs on their basis and to perform comparative study of HIV-specific T cell responses mediated by these vaccines.

To produce novel HIV polyepitope antigens we have used experimentally validated HIV-1 CTL and T-helper epitopes extracted from Los-Alamos HIV Immunology Database. To choose the most promiscuous HLA-binders and to design polyepitope immunogens we used our previously developed original software TEpredict and PolyCTLDesigner (http://tepredict.sourceforge.net) [32]. To enhance the processing and presentation efficiency of our antigenic constructs we included additional sequences in their structures. These are spacer amino-acid residues that optimize proteasomal/immunoproteasomal processing (p-imp flank) of polyepitope construct and TAP transport (tap flank) of liberated peptides; N-terminal ubiquitin; N-terminal signal peptide (signal sequence of E3/gp19K protein of adenoviruses), and C-terminal tyrosine motif of LAMP-1 protein. According to theoretical design, we constructed three DNA-vaccines, encoding novel T-cell immunogens TCI-N, TCI-N2, and TCI-N3, which should undergo MHC class I and/or MHC class II epitope presentation pathways to generate HIV-specific CD8+ and CD4+ T-lymphocyte responses. In the present study we demonstrated the capability of artificial polyepitope HIV-1 immunogens to induce HIV-specific T cell response and assessed the influence of additional signals in the immunogen structures on the magnitude of CD4+ and CD8+ HIV-specific responses.

## Materials and Methods

### Synthesis of the genes encoding target polyepitope immunogens

Artificial gene sequences encoding target immunogens TCI-N, TCI-N2, and TCI-N3 have length of 2451, 2532, and 2679 bp, respectively. Design of gene sequences was done using different software and online services (DNASTAR, Vector NTI, NCBI-BLAST, etc.) that enable to optimize gene sequences for their high expression in human cells. Kozak sequence (CCGCCACC) was added at the 5'-end of the DNA sequence before the start codon ATG. Three stop codons (TAGTGATGA) were added at the 3'-end of immunogen sequences. Designed genes were synthesized using the ligating method based on thermally stable RNA-ligase (NEB, USA) with subsequent PCR and cloning into pAL-TA vector. Oligonucleotide synthesis was carried out using ABI3900 synthesizer (Applied Biosystems, USA). While synthesizing, modified protocol was used that enabled to obtain oligonucleotides up to 90 bp in length and of 95% purity. Validity of synthesized sequences was confirmed after its subsequent sequencing with an automated sequencer ABI3730x1.

### Target genes cloning and plasmid construction

Genes encoding polyepitope immunogens TCI-N, TCI-N2, and TCI-N3 were cloned into the pcDNA3.1/*Myc*-His(+)/*lac*Z (Invitrogen, USA) plasmid vector. As a result three target plasmids P1, P2, and P3 were obtained. Sequences of cloned genes and plasmid fragments containing the promoter region were verified by sequencing.

### Production and purification of plasmid DNA for immunization

All DNA plasmids were purified with a Wizard PureFection Plasmid DNA Purification System Endotox Free (Promega, USA) according to the manufacturer’s protocol.

### Transfection and cell cultures

293T (human embryonic kidney cell line) cells were cultured in RPMI (Biolot, Russia) supplemented with 10% fetal bovine serum (FBS, Gibco-BRL, Rockville, MD).

DNA plasmids P1, P2, and P3 were transfected into 293T cells using the Lipofectamine (Invitrogen, USA) according to the manufacturer’s recommendation. Cells were harvested for 48 hours before the protein expression was investigated.

### Evidence of the target gene expression in vitro

Target genes expression was evaluated *in vitro* using three methods:

1. Reverse transcription-PCR was performed to determine specific polyepitope immunogen (polyE) mRNA.
  Total RNA from 1×10^6^ transfected 293Т cells was extracted using “GE Healthcare RNAspin Mini RNA isolation Kit” according to instructions recommended by the manufacturer. The DNase-treated RNA samples were converted to cDNA using standard random primers 6 nucleotides length with Sensiscript RT Kit (Qiagen). PCR was performed with two pairs of specific primers on the region of the polyE gene using TaqDNA polymerase (Invitrogen, USA). PCR-amplified DNA fragments have been electrophoresed on 2.5% agarose gel.
2. SDS-PAGE and Western immunoblotting assay
  Proteins from DNA-transfected cell lysates were separated on 15% SDS-PAGE gel and then transferred onto nitrocellulose membranes using a semidry gel electroblotter (LKB). Immunodetection of polyepitope proteins expression was performed with the SNAP i.d. Protein Detection System (Millipore, USA). After blocking filters overnight with 3% bovine serum albumin (AMRESCO LLC, USA) the membranes were incubated with 1 μg/ml of anti-mouse monoclonal 29F2 antibodies (VECTOR-BEST, Russia) followed by a peroxidase-conjugated secondary antibody. Monoclonal 29F2 antibodies specifically bind to “marker” epitope EPFRDYVDRFYKTL from Gag which was included in C-terminus of all immunogens and thus provides the monitoring of the expression of target genes. As control, the membranes were probed with аnti-beta аctin antibody (Abcam, UK) at a dilution of 1:3,000.
3. Indication of polyepitope protein expression using 29F2-FITC monoclonal antibodies
  Transfected 293T cells were permeabilized with Cytofix/Cytoperm Plus Fixation/Permeabilization Kit (BD Biosciences), washed twice with PBS (phosphate buffered saline) and stained 30 min on ice in the dark with FITC-labeled 29F2 monoclonal antibodies (VECTOR-BEST, Russia) against “marker” epitope EPFRDYVDRFYKTLR from HIV-1 p24 Gag protein which was included in C-terminus of all immunogens. A total of 10^5^ stained cells were collected on the flow cytometer FACSCalibur (Becton Dickinson) and were analyzed using Cell Quest software.

### Immunization of experimental animals and collection of samples

**Fig 1:**
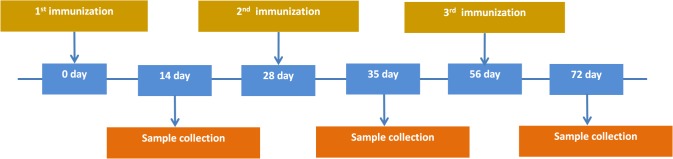
Immunization schedule.

The immunogenicity of engineered DNA vaccine constructs P1, P2, and P3 has been evaluated on BALB/c mice. All animal procedures and care were approved by the IACUC (Institutional Animal Care and Use Committee) of the State Research Center of Virology and Biotechnology Vector. Groups of 5- to 6-wk-old female BALB/c mice (N = 10 mice per group) were vaccinated once, twice or three times according to schedule (Fig. 1) by intramuscular injection 100 μg DNA. An equivalent dose of vector plasmid pcDNA3.1 was used for the control group of mice. Mice were sacrificed at various times after the immunization (Fig. 1). Spleens were collected from the individual animals from each of the vaccinated groups and negative control group. Splenocytes were isolated by pressing spleens individually through a cell strainer (BD Falcon) using a 2-ml syringe rubber plunger. Following the removal of red blood cells with RBC Lysis Buffer (Sigma), splenocytes were washed and resuspended in RPMI 1640 supplemented with 10% FBS, penicillin/streptomycin.

### Intracellular cytokine staining and flow cytometry

One million splenocytes collected from immunized mice were stimulated with mix of synthetic HIV peptides corresponding to the mouse H-2^d^ restricted epitopes encoded by the DNA vaccine constructs (Table 1) and incubated for 20 hours at 37°C, 5% CO2 and additionally for 5 h with Golgi Plug (BD Biosciences). Cells were washed with PBS and permeabilized with Cytofix/Cytoperm Plus Fixation/Permeabilization Kit (BD Biosciences). Perm/Wash buffer was used to wash cells before staining with following monoclonal antibodies: PerCP Rat Anti-Mouse CD4, FITC Rat Anti-Mouse CD8a, PE Hamster Anti-Mouse CD3ε, APC Rat Anti-Mouse IFN-γ, APC Rat Anti-Mouse IL-2 (BD Pharmingen). A total of 10^5^ gated events were collected by FACSCalibur using Cell Quest software.

**Table 1:** HIV peptides restricted by mouse BALB/c MHC class I molecules (H-2^d^) which were used for mouse splenocytes stimulation.

**Epitope (peptide) sequences** * ^)^	**Protein (amino-acid residues)**
AMQMLKETI	p24(65–73)
IFQSSMTKI	RT(159–167)
EPFRDYVDRF	p24(159–168)
VYYDPSKDLI	RT(317–326)
SYHRLRDFI	gp160(767–775)
SLYNTVATL	p17(77–85)

*^)^The sequences of these peptides are the exact copies of mouse H-2^d^ restricted epitopes encoded by the DNA vaccine constructs.

### Statistical analysis

Statistical analysis was performed with pairwise comparison using either one- or two-sided Mann-Whitney U test implemented in R statistical software [33]. Multiple testing p-value correction was performed with FDR procedure [34]. The differences between two groups were considered to be significant when the adjusted *P* value was ≤ 0.05.

## Results

### Design of optimized artificial HIV-1 poly-T cell-epitope immunogens

**Choice of the CD8+ T cell epitopes for inducing the CD8+ T cell responses.** To construct new HIV polyepitope immunogens known HIV-1 CTL epitopes were taken from the list of best-defined HIV CTL/CD8+ epitopes from HIV molecular immunology database (http://www.hiv.lanl.gov/content/immunology/tables/optimal_ctl_summary.html). Epitopes were selected according to their conservation in HIV-1 isolates of clades A, B, and C. Peptides with conservation rate more or equal to 80% in at least one clade were selected for further analysis. Then we have predicted additional HLA-binding specificity for selected HIV-1 CTL epitopes. This was done to choose the most promiscuous HLA-binders and at the same time to cover the most frequent HLA alleles.

Binding of oligopeptides to HLA molecules was predicted using our original software TEpredict [35]. TEpredict uses PLS (partial least squares) regression models trained with quantitative peptide:HLA binding data for 35 most frequent HLA-A and HLA-B alleles obtained from Immune Epitope Database [36]. Taking amino acid sequence of the peptide and an HLA allele name as inputs the program predicts an affinity of their interaction. Peptide is considered to be a binder, and to be a potential epitope, if predicted pIC_50_ value is grater or equal to 6.3 (IC_50_ ∼ 500 nM). More detailed description of the program and its capabilities can be found in the paper [35] and at the project web-site (http://tepredict.sourceforge.net). Comparative testing with several other T-cell epitope prediction tools confirmed the high accuracy of TEpredict, additional results can be found at http://tepredict.sourceforge.net/comparison/.

Further selection of CTL epitopes was performed with our PolyCTLDesigner program [32] aimed for rational design of polyepitope immunogens. PolyCTLDesigner can also be used to choose the minimal set of epitopes covering selected MHC repertoire with desired rate of redundancy. Here CTL epitopes were selected to cover the most of 35 HLA alleles (included into TEpredict) with at least five-fold excess—using the greedy search algorithm the program found the minimally sized subset of epitopes covering the whole HLA repertoire (selected), this procedure was repeated 5 times.

In total 50 CTL epitopes were selected for design of target immunogens (Table 2). They are (i) derived from the main virus proteins Env, Gag, Pol, Nef, and Tat; (ii) conserved among HIV-1 clades A and/or B and/or C; and (iii) cover a wide range of the most common allelic variants of HLA molecules.

**Table 2:** Predicted CD8+ epitopes in the sequences of HIV-1 proteins Env, Gag Pol, Nef, and Tat.

**Epitope**		**The efficiency of epitopes (peptides) interaction (affinity) with MHC molecules** ** ^)^																																
IDs*^)^	**Sequence**	A*0101	A*0202	A*0203	A*0206	A*0301	A*1101	A*2301	A*2402	A*2403	A*2601	A*2902	A*3001	A*3002	A*3101	A*6801	A*6802	A*6901	B*0702	B*0801	B*1501	B*1801	B*2705	B*3501	B*4001	B*4002	B*4402	B*4403	B*4501	B*5101	B*5301	B*5401	B*5701	B*5801
16484_POL_0.99_0.95_0.98	**GKLNWASQI**										6.87											7.79					6.85	7.31						
2435_GAG_0.87_0.89_0.84	**FSPEVIPMF**										8.88			7.32									6.84											
4776_GAG_0.98_0.98_0.97	**GLNKIVRMY**											7.44		7.39							6.82													
38481_POL_0.95_0.93_0.94	**KTAVQMAVF**										7.04			7.47												7.05								6.84
32421_GAG_0.84_0.86_0.90	**KRWIILGLN**					8.17	6.81						6.87	7.56								7.40										7.54		
34944_POL_0.99_0.95_0.98	**LVGKLNWAS**	6.81					7.65																	7.70										
43418_ENV_0.70_0.89_0.56	**VYYGVPVWK**						7.66						7.24		7.07	7.88										6.89								
9209_POL_0.92_0.91_0.92	**ETFYVDGAA**										7.23			6.93		7.62	7.32	7.64				7.10		7.20								8.71	7.13	
134586_ENV_0.82_0.75_0.81	**FCASDAKAY**											7.70		7.73										8.00										
13905_GAG_0.96_0.23_0.96	**VRMYSPVSI**																					7.60					7.02	6.95	7.15					
43968_GAG_0.09_0.88_0.85	**TPQDLNTML**										7.53			6.84					6.95							7.36								
36952_POL_0.93_0.92_0.92	**IVTDSQYAL**		7.97								6.88			6.89				6.85						7.82		8.16								
2181_GAG_0.86_0.81_0.04	**EVIPMFSAL**		7.12	7.24	7.61				7.57		7.19						7.41	7.33				6.91		7.78		7.31								
32589_POL_0.97_0.90_0.94	**AVFIHNFKR**						8.48						7.15		7.98	7.34																		
94381_ENV_0.88_0.83_0.82	**LFCASDAKA**		6.85																					7.01								6.99		6.91
8741_POL_0.96_0.95_0.98	**TVLDVGDAY**										8.39	7.26		7.59		6.91					6.83	7.28		7.10			6.87						6.87	
734_POL_0.96_0.92_0.92	**TPVNIIGRN**						6.93																								7.23	6.83		
4062_GAG_0.93_0.92_0.95	**SPRTLNAWV**													7.20					7.33							7.55						6.84		
4201_POL_0.95_0.92_0.97	**TDSQYALGI**																	7.71				7.32				7.99	7.06	7.38	7.40		6.92			
17520_POL_0.37_0.90_0.95	**VIYQYMDDL**		7.20						7.07	8.35	7.08															6.92								6.99
616_VIF_0.08_0.82_0.01	**WHLGQGVSI**				7.39			6.93														7.88			7.36	9.14	6.88			7.75				
25479_GAG_0.01_0.69_0.82	**VQNLQGQMV**																												6.87			8.04		
17755_POL_1.00_0.98_0.98	**KLVDFRELN**					6.85							6.89	7.10																				
2359_POL_0.84_0.70_0.17	**ETKLGKAGY**										7.07			7.35																				
34965_POL_0.77_0.91_0.86	**KIQNFRVYY**											8.91	7.11		7.30											7.89							6.84	
38635_POL_0.73_0.84_0.95	**ITLWQRPLV**										7.54													6.96		8.15			7.04			8.97		
26003_GAG_0.72_0.81_0.69	**MREPRGSDI**				6.83																					7.79						7.52		
2443_GAG_0.97_0.97_0.97	**GHQAAMQML**		6.91	6.87						7.00																8.63								
27316_GAG_0.57_0.01_0.83	**YVDRFFKTL**	6.94								6.87	7.07									6.82						7.32								
9405_GAG_0.73_0.93_0.85	**WASRELERF**											6.98		7.82										7.25		8.42								7.45
26064_POL_0.95_0.92_0.95	**VTDSQYALG**					6.90	7.53							7.33																				
31240_GAG_0.66_0.88_0.80	**AFSPEVIPM**		7.22	7.53	7.48					7.34				6.91										7.46		7.25								
146630_ENV_0.93_0.97_0.95	**TVYYGVPVW**						7.08							6.94		7.08										7.87							6.81	
21393_GAG_0.01_0.91_0.00	**YVDRFYKTL**	7.12									7.11															7.47								
35328_POL_0.85_0.06_0.77	**GKKAIGTVL**																		6.88							7.27								
8085_GAG_0.72_0.83_0.78	**IRLRPGGKK**																					7.38	7.00											
2700_GAG_0.75_0.82_0.82	**RQANFLGKI**			7.27	7.44				6.85		7.03							7.74								6.95						7.15		
7351_POL_0.93_0.83_0.93	**IYQEPFKNL**									7.95																								
1035_GAG_0.03_0.83_0.01	**ETINEEAAE**															7.50		6.95				7.26		7.56										
7003_NEF_0.82_0.74_0.77	**RPQVPLRPM**													7.17					7.88															
35713_POL_0.63_0.84_0.84	**ETPGIRYQY**													6.88																			6.85	
28077_POL_0.99_0.94_0.98	**KLNWASQIY**	6.97								7.61		7.55									7.46													
22847_GAG_0.58_0.01_0.83	**DYVDRFFKT**								7.17									7.91																
27934_GAG_0.88_0.86_0.90	**TINEEAAEW**									7.43		7.88		6.96																			6.85	7.58
28759_GAG_0.77_0.92_0.87	**SEGATPQDL**													7.21												8.26	6.94							
24079_GAG_0.01_0.94_0.00	**RDYVDRFYK**					7.10	7.83						8.30		7.88							7.31				7.82								
14241_GAG_0.94_0.01_0.94	**RDYVDRFFK**					7.43	8.06						8.61	7.44	7.70							7.27				8.17								
10794_GAG_0.90_0.77_0.90	**CRAPRKKGC**			6.99																			6.89			7.23			6.86					
340_TAT_0.95_0.74_0.86	**KGLGISYGR**														7.25	6.90		6.92				7.43									7.79			
3149_GAG_0.01_0.91_0.00	**DRFYKTLRA**																	7.21				7.23				7.21						7.43		

*^)^ Epitope IDs are written in the format 16484_POL_0.99_0.95_0.98, where the separated position means: Peptide number_Protein_Conservatism in A clade_Conservatism in B clade_Conservatism in C Clade.

**^)^ The efficiency of epitopes (peptides) interaction (affinity) with MHC molecules represents values of pIC50: pIC50 ≥ 7.3—high affinity peptides (red and brown); 6.3 ≤ pIC50 < 7.3—moderate affinity peptides (yellow); pIC50 < 6.3—low affinity peptides.

**Polyepitope HIV-1 immunogen sequence design.** Design of target T-cell immunogens was carried out using PolyCTLDesigner software [32]. PolyCTLDesigner selects superior spacers for each pair of epitopes (to provide proteasomal release of the epitopes and to optimize binding of generated peptides to TAP) and chooses optimal arrangement of epitopes within designed construction to increase efficiency of polyepitope processing and favor target epitopes presentation. It also tries to minimize the number of undesired non-target junctional epitopes. Graph theory approach is used to construct optimal polyepitope immunogen: PolyCTLDesigner produces weighted directed graph with nodes corresponding to peptides (epitopes) and edges corresponding to epitope matchings. Each edge has two parameters: the optimal spacer sequence and its weight calculated by the ranking function taking into account the efficiency of proteasomal cleavage site, the number of junctional epitopes, the length of the spacer etc. The best junction has the lowest weight, and the sequence of desired polyepitope antigen is determined as the least weighted complete simple path in the graph. To predict proteasomal and/or immunoproteasomal cleavage PolyCTLDesigner uses models developed by Toes et al. [37]. Peptide binding to TAP is predicted with models developed by Peters et al. [36].

All three novel T-cell immunogens TCI-N, TCI-N2, and TCI-N3 contain a “core” sequence—polyE *(polyEpitope)*. polyE sequence consists of two fragments—polyCTL and polyTh, the first of which contains a sequence of CD8+ CTL-epitopes and the second—the sequence of CD4+ Th-epitopes.

To investigate the immunogenicity of the vaccine constructs both in humans and in BALB/c mice eight additional “marker” CD8+ CTL-epitopes restricted by both human and mouse BALB/c MHC class I molecules were included in polyCTL part of polyE. These epitopes are presented as a separate fragment (VYYDPSKDLI-ADL-SYHRLRDFI-ADG-TPLCVSLSF-AIAV-KLTPLCVTL-AMQMLKETI-AD-SLYNTVATL-ALYNTVATLQ-RDLS-IFQSSMTKI), and its sequence was also optimized for proteasomal/immunoproteasomal processing using PolyCTLDesigner software. These epitopes were selected from the list of experimentally-verified HIV CTL/CD8+ epitopes (http://www.hiv.lanl.gov/content/immunology/tables/ctl_summary.html).

To design polyTh fragment of polyE sequence we used six highly conserved HLA-DR binding CD4+ T-helper peptides (KTAVQMAVFIHNFKR, KRWIILGLNKIVRMY, SPAIFQSSMTKILEP, WEFVNTPPLVKLWYQ, HSNWRAMASDFNLPP, QKQITKIQNFRVYYR), for which in the case of HIV-1 clade A predicted conservatism was at least 40%. Epitopes were selected from the list of experimentally-verified HIV T-Helper/CD4+ epitopes (http://www.hiv.lanl.gov/content/immunology/tables/ctl_summary.html). In addition, universal T-helper peptide 'PADRE' (**pa**n HLA **DR**-binding **e**pitope—AKFVAAWTLKAAA) that is highly immunogenic CD4+ T cells restricted by numerous mouse and human class II allomorphs [38] was included in the T-helper fragment. Mentioned peptide induces CD4+ T-helper responses in association with multiple HLA-DR-allomorphs and with murine MHC class II molecules. Assembly of T-helper epitopes in the polyepitope construction was performed by R/K-R/K motifs, which represent cleavage sites for lysosomal cathepsins B and L [30,31].

To estimate expression and metabolic stability of designed immunogens EPFRDYVDRFYKTLR epitope from HIV-1 p24 Gag protein was added to the C-terminus of the polyepitope construct, which is recognized by 29F2 monoclonal antibodies (mAb) and allows further detection of polyepitope immunogen expression using mAb, conjugated with fluorescent tag.

**Fig 2:**
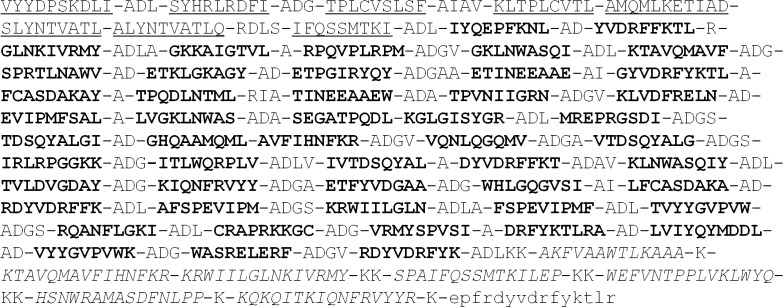
The “core” amino-acid sequence of polyepitope T cell immunogen. Here CD8+ CTL-epitopes restricted by both human (HLA A*02) and inbred mice BALB/c (H-2^d^) MHC class I molecules are underlined; CD8+ CTL-epitopes restricted by human MHC class I molecules are bold; CD4+ T-helper epitopes restricted by human MHC class II molecules are italic; Gag epitope which is recognized by 29F2 monoclonal antibodies marked with small letters (epfrdyvdrfyktlr).

Thus, resulting polyE sequence has the following structure (Fig. 2).

**Enhancing epitope processing and presentation.** To ensure the efficient processing and presentation of target CD8+ CTL and CD4+ T-helper epitopes we included additional sequences in the structure of polyepitope immunogens:

- N-terminal ubiquitin (Ub) with substitution of the C-terminal Gly to Val to prevent liberation of Ub cleavage by Ub hydrolases [39]
- (MQIFVKTLTGKTITLEVEPSDTIENVKAKIQDKEGIPPDQQRLIFAGKQLEDGRTLSDYNIQKESTLHLVLRLRGV_76_);
- N-terminal signal peptide (in our case MRYMILGLLALAAVCSAA—the signal sequence of the adenovirus protein E3/gp19K);
- C-terminal tyrosine-based motif of LAMP-1 glycoprotein (RKRSHAGYQTI).

N-terminal ubiquitin targets the polyepitope construct to the proteasome for degradation and subsequent binding of released peptides to MHC class I molecules. N-terminal signal peptide provides immunogen delivery to the endoplasmic reticulum and to the secretory pathway, and tyrosine-based motif of LAMP-1 glycoprotein directs immunogen from the secretory pathway to lysosomes for degradation, where the resulting peptide fragments bind to MHC class II molecules for presentation to CD4+ T-lymphocytes [40,41].

**Fig 3:**
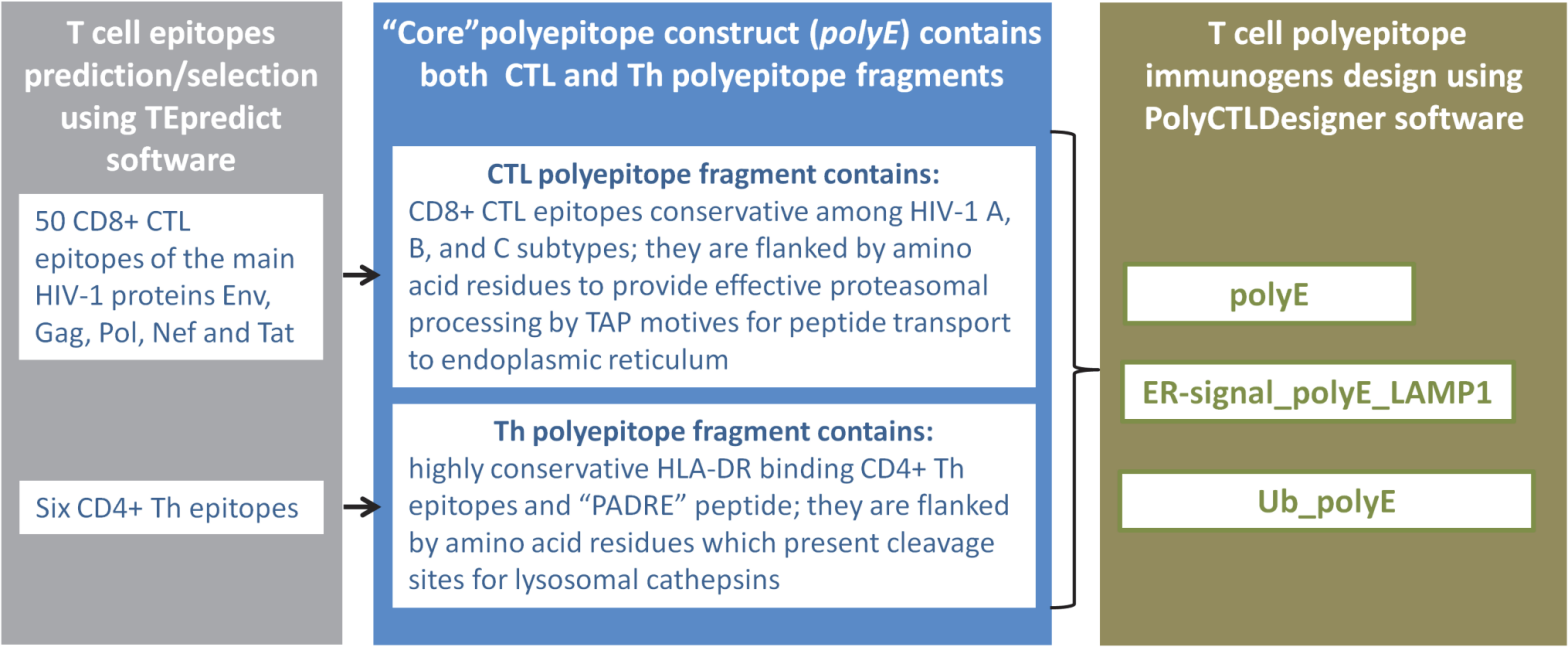
Design of T cell polyepitope immunogens TCI-N (polyE), TCI-N2 (ER-signal_polyE_LAMP1), and TCI-N3 (Ub_polyE).

According to the proposed design N- and C-terminal sequences were added to polyE construct in three different combinations. As a result we obtained three different structures of designed T cell immunogens—TCI-N, TCI-N2, and TCI-N3 which provide MHC class I and MHC class II pathways of target polyepitope immunogen presentation to CD8+ and CD4+ T-lymphocytes (Fig. 3).

### Target genes cloning and plasmid construction

Genes encoding polyepitope immunogens TCI-N, TCI-N2, and TCI-N3 were cloned into the pcDNA3.1/*Myc*-His(+)/*lac*Z (Invitrogen, USA) plasmid vector. As a result three target plasmids P1, P2, and P3 were obtained:

- P1: pcDNA_Kozak_polyE (TCI-N);
- P2: pcDNA_Kozak_ER-signal_polyE_LAMP-1 (TCI-N2);
- P3: pcDNA_Kozak_Ub_polyE (TCI-N3).

### In vitro expression of genes encoding target polyepitope proteins

**Fig 4:**
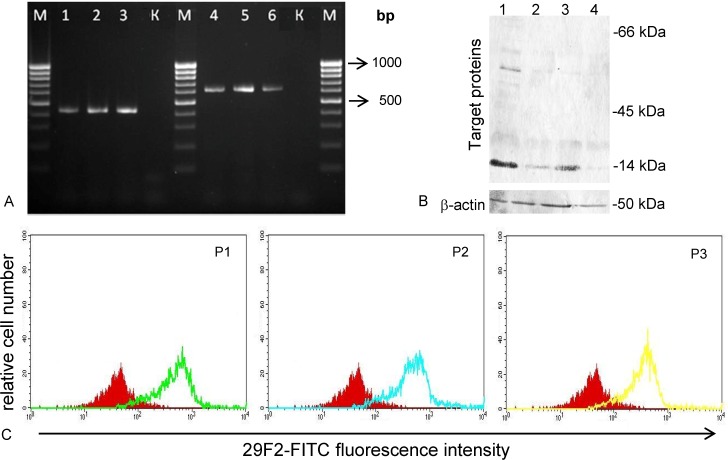
Expression of genes encoding artificial polyepitope proteins TCI-N, TCI-N2, and TCI-N3. (A) 2.5% agarose gel electrophoresis of RT-PCR products derived from isolated mRNA after 293T cells transfection with obtained plasmids. Lanes 1–3, PCR fragments of 435 bp correspond to P1, P2, and P3 plasmids; Lanes 4–6, PCR fragments of 624 bp correspond to P1, P2, and P3 plasmids; K, negative control sample (total mRNA extracted from transfected cells); M, 100bp DNA Ladder (SibEnzyme, Russia). (B) Western blot analysis of expression products of genes encoding TCI-N, TCI-N2, and TCI-N3 proteins in transfected 293T cells. Lanes P1, P2, P3 are lysates of 293T cell transfected with P1, P2, P3, respectively; lane К represents lysates of 293T cells transfected with vector plasmid pcDNA3.1. The membrane containing the cell-associated fractions was also probed with аnti-beta аctin antibody to control for equal loading of the samples. (C) Flow cytometry analyses of expression products of target genes after transfection of 293T cells with P1, P2, and P3 plasmids. Histogram overlays of target proteins stained with FITC-labeled 29F2 monoclonal antibodies (green peak, TCI-N; blue peak, TCI-N2; and yellow peak, TCI-N3 polyepitope protein expression). Negative control (read peak) represents 293T cells transfected with vector plasmid pcDNA3.1.

To assess gene expression of obtained DNA constructs in mammalian cells, 293T cells were transfected *in vitro* with plasmid DNA molecules P1, P2, and P3. Reverse transcription-PCR was used to detect specific immunogen polyE mRNA, and it was shown that all three plasmids expressed polyE transcripts (Fig. 4A), because all of them contained polyE construct (Fig. 4A).

Western immunoblotting assay was performed to confirm the presence of expression products of genes encoding TCI-N, TCI-N2, and TCI-N3 immunogens in 293T transfected cells. Using 29F2 monoclonal antibodies to “marker” epitope EPFRDYVDRFYKTLR from HIV-1 p24 Gag protein we demonstrated that 293T cells transfected with P1, P2, and P3 plasmids contained expression products of target genes (Fig. 4B). However, the majority of specific proteins were detected preferentially in the low molecular weight region (14 kDa). This may indicate that target proteins undergo processing in cells due to incorporated sites for proteolysis which flank antigenic peptides inside polyepitope construct and leads to instability of the full-length target protein.

Since we could not detect full-length target proteins using Western immunoblotting and relatively high background, to confirm expression of target genes we used more sensitive flow cytometry assay using FITC-labeled 29F2 monoclonal antibodies (Fig. 4C). Presence of peak of specific fluorescence in FL1 shows that DNA vaccine constructs P1, P2, and P3 provide expression of target proteins in eukaryotic cells.

### The artificial polyepitope T cell immunogens induced HIV-specific CD4+ and CD8+ T cell immune responses

The primary goal of our study was to determine whether artificial polyepitope T cell immunogens are able to elicit HIV-specific CD4+ and CD8+ T cell immune responses. Experimental groups of BALB/c mice were immunized three times intramuscularly with P1, P2, and P3 DNA-vaccines encoding target immunogens TCI-N, TCI-N2, and TCI-N3, respectively (n = 10 mice per group). Animals of control group were immunized with empty vector plasmid pcDNA3.1.

**Fig 5:**
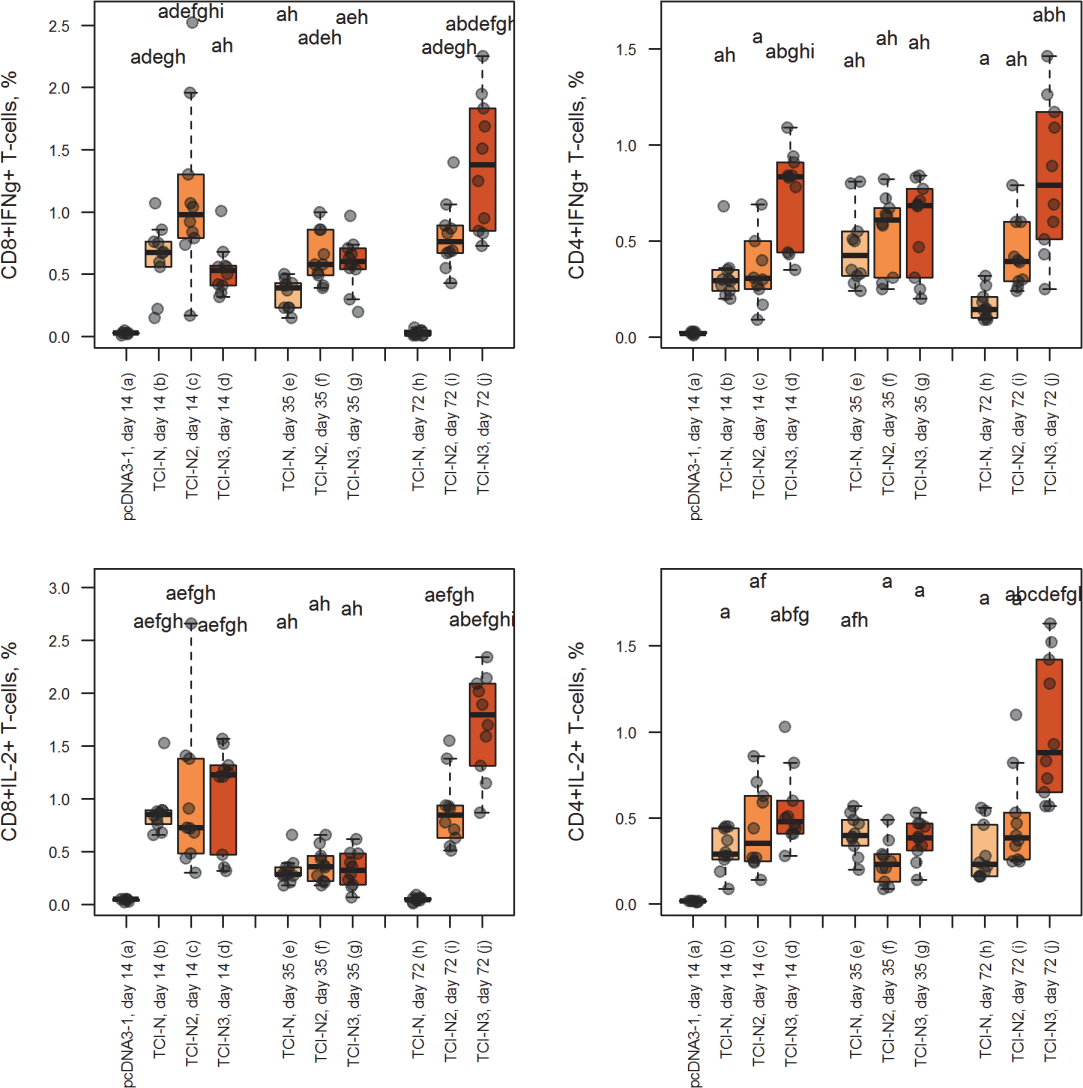
The magnitude of HIV-1-specific CD4+ and CD8+ T lymphocyte responses following immunization with polyepitope T cell immunogens TCI-N, TCI-N2 and TCI-N3. Groups of BALB/c mice (n = 10 per group) were vaccinated once, twice or three times by intramuscular injection of plasmids P1, P2, P3 at day 0, 28, and 56. Immunization with vector plasmid pcDNA3.1 was performed as control at day 0. Box-and-whisker plots depict the magnitude of responses in each group for each time point (*P* value was ≤ 0.05). Levels of cytokine-producing T-lymphocytes are depicted on the *y* axis. Individual groups of animals are depicted on the *x* axis.

The ability of CD4+ and CD8+ T cells to express IL-2 and IFN-γ was tested by ICS after *in vitro* stimulation of splenocytes collected from immunized mice with mix of synthetic HIV peptides corresponding to selected mouse H-2^d^ restricted epitopes encoded by the DNA vaccine constructs (see Table 1). We observed that all groups of mice immunized with DNA-vaccines encoding polyepitope immunogens developed HIV-specific CD4+ and CD8+ T cell responses. In groups of mice immunized with recombinant plasmids encoding artificial immunogens levels of IL-2 and IFN-γ producing T-lymphocytes were significantly higher than in control group administered with pcDNA3.1 (Fig. 5). Thus, we demonstrated that DNA vaccine constructs expressing artificial HIV-1 polyepitope T cell immunogens induced CD4+ and CD8+ T cell responses following BALB/c mice immunization.

### The immunogens with ubiquitin and LAMP-1+ER-signal sequences induced higher magnitude of HIV-specific CD4+ and CD8+ T cell responses compared to the immunogen without additional signaling sequences

The second aim was to study the influence of additional N- and C-terminal sequences, such as ubiquitin and LAMP-1+ER-signal, on the magnitude of HIV-specific CD4+ and CD8+ T cell responses. Groups of BALB/c mice (n = 10 in each group) received three injections of DNA vaccine plasmids according to immunization protocol. To assess immunogenicity of DNA vaccines encoding TCI-N, TCI-N2, and TCI-N3 immunogens we measured levels of IL-2 and IFN-γ producing CD4+ and CD8+ T cells by ICS during several time points after immunization. After three injections of DNA-vaccines we found that DNA-vaccine constructs encoding TCI-N3 and TCI-N2 immunogens (containing ubiquitin and LAMP-1+ER-signal, respectively) showed statistically significant growth of the magnitude of HIV-specific CD4+ and CD8+ T cell responses in comparison with construct expressing TCI-N immunogen (without additional signaling sequences) (Fig. 5). Thus, we have shown that N- and C-terminal sequences such as ubiquitin or LAMP-1+ER-signal improve the immunogenicity of polyepitope DNA-vaccines in our study.

### The polyepitope immunogen containing ubiquitin sequence induced higher magnitude of T cell responses compared to the immunogen containing LAMP-1+ER-signal sequences

The third objective of our study was to determine which of the three DNA-vaccine constructs elicits greater magnitude of HIV-specific CD4+ and CD8+ T cell responses. We have found that after the third immunization DNA-vaccine construct encoding TCI-N3 immunogen (Ub_polyE) containing N-terminal ubiquitin induced larger numbers of IL-2 and IFN-γ-producing CD4+ and CD8+ T cells (p ≤ 0.01) compared to DNA-vaccine encoding ER-signal_polyE_LAMP-1 immunogen (Fig. 5). Thus, DNA-vaccine construct encoding TCI-N3 immunogen (Ub_polyE) generates the most significant levels of vaccine-mediated expression of IL-2 and IFN-γ by CD4+ and CD8+ T-lymphocytes in our study after three immunizations.

## Discussion

Eliciting an immune response that can protect against the great diversity of circulating HIV-1 strains is a formidable challenge [42]. The polyepitope strategy can be a promising approach in the field of T cell-based vaccine development [15]. This strategy is based on investigator-designed artificial genes expressing either in DNA plasmid or in a viral vector a chain of epitopes combined in a single artificial vaccine construct.

Ideally, such polyepitope T cell vaccines should induce CD8+ and CD4+ T-lymphocyte responses against each epitope included in their structure. Progress in the identification of T cell epitopes as well as in understanding of mechanisms of MHC class I and MHC class II pathways of antigen processing and presentation provided a basis for rational design of polyepitope vaccines inducing efficient T cell responses against HIV-1 and other viruses [43–45].

Previously we described design and comparison of experimental DNA vaccines comprising ten selected HLA-A2 epitopes from HIV-1 antigens Env, Gag, Pol, Nef, and Vpr [5]. Using HLA-A2-transgenic mice we compared a number of parameters including different strategies for targeting polyepitope constructs to the ubiquitin-proteasome pathway and including different combination of spacer sequences between epitopes to provide their proteasome liberation and TAP transport. It was demonstrated that the most immunogenic vaccine construct contained the N-terminal ubiquitin for targeting the polyepitope to the proteasome and included both proteasome liberation and TAP-transport spacer sequences that flanked the epitopes within the polyepitope construct. The immunogenicity of determinants was strictly related to their affinities for HLA-A2. Although that construct was the most immunogenic, we cannot say that it was the optimal one, since when designing polyepitope constructs we did not optimize the order of epitopes and the structure of spacer sequences. Nevertheless, obtained results support the concept of rational vaccine design based on the knowledge of antigen processing. In fact, these findings have provided the theoretical basis for our software PolyCTLDesigner, intended for designing optimal polyepitope antigens [32].

In this study we used TEpredict and PolyCTLDesigner software to design new polyepitope T cell immunogens TCI-N, TCI-N2, and TCI-N3 comprising multiple experimentally-verified CD8+ CTL- and CD4+ Th-epitopes from HIV-1 proteins Env, Gag, Pol, Nef, and Tat.

To be effective, T cell-based vaccine may focus the cellular immune responses on the most conserved epitopes which are restricted by the most common HLA class I molecules [46], since conserved viral antigens can play a crucial role in protection against wide variety of HIV-1 antigenic variants [11,46–48]. Furthermore, optimal CD8+ T cell vaccines should stimulate CD8+ CTL responses against multiple HIV-1 antigenic determinants [11].

In addition, construction of HIV-1 T cell-based vaccine requires that polyepitope immunogen should induce CD8+ CTL responses restricted by different HLA alleles to be able to cover genetic polymorphism of HLA class I antigens at a population level. It is known that optimally selected HIV-1 epitopes restricted by five different HLA class I alleles cover from 80 to 90% of population; and nine peptides are needed to cover the general population [13,49,50]. At the same time it was shown that CD8+ CTL-epitopes with high binding affinity to MHC class I molecules and with cross-reactivity for multiple MHC molecules positively correlated with vaccine immunogenicity [14].

So, from the lists of experimentally-verified HIV CTL/CD8+ epitopes we selected peptides conserved in at least 80% of HIV-1 isolates of either clades A, B or C. Using TEpredict software we have also predicted additional HLA-binding specificity for these peptides to choose the most promiscuous HLA-binders. To design polyE immunogen we chose a minimum set of optimal epitopes that have a high or medium affinity to MHC class I molecules and cover a variety of MHC class I alleles for 11 geographic populations. To design polyTh fragment of polyE sequence we used six highly conserved HLA-DR binding CD4+ T-helper peptides selected from the list of experimentally-verified HIV T-Helper/CD4+ epitopes. As a result 50 CD8+ CTL and 6 CD4+ T-helper epitopes were selected for design of “core” polyepitope immunogen sequence—polyE.

When designing polyE sequence with the use of PolyCTLDesigner we considered such factors, as efficiency of proteasomal/immunoproteasomal processing of polyepitope protein and peptide interaction with TAP. For this purpose all epitopes were flanked (if necessary) by sequences optimizing processing of polyepitope construct and presentation of released peptides.

To ensure HIV-specific CD4+ and CD8+ T cell responses we used a series of approaches. Since cytoplasmic degradation of newly synthesized viral proteins proceeds via ubiquitin-dependent pathway involving proteasomes, one of the promising approaches to enhance CD8+ CTL responses is based on specific targeting of vaccine antigens to proteasome by attachment of ubiquitin [24]. The resulting peptides are then transported by the TAP1/TAP2 heterodimers into endoplasmic reticulum where they associate with MHC class I molecules and β-microglobulin [51]. Therefore, to enhance CD8+ CTL responses a construct with N-terminal ubiquitin was developed [52]. As far as polyepitope T cell immunogen comprises CD4+ Th-epitopes, we assumed that the potential of obtained DNA-vaccine could be increased by targeting of polyE immunogen to lysosome for MHC class II antigen processing and presentation pathway [53,54]. For this purpose we used two sequences to flank polyepitope construct: N-terminal signal peptide (ER-signal) and C-terminal tyrosine motif of LAMP-1 protein. N-terminal signal peptide provides immunogen delivery to the endoplasmic reticulum and to the secretory pathway, and LAMP-motif directs this immunogen from the secretory pathway to lysosomes for liberation of CD4+ Th-epitopes and their presentation to CD4+ T-lymphocytes.

As a result three polyepitope constructs were developed: TCI-N (*polyE*), TCI-N2 (ER-signal_*polyE*_LAMP-1), and TCI-N3 (Ub_*polyE*) providing different strategies of target polyepitope immunogen presentation (MHC class I and MHC class II) pathways.

Polyepitope constructs described in the manuscript were designed for humans. However, before conducting costly studies of immunogenicity of developed immunogens in human PBMCs *ex vivo*, it was necessary to show that constructed immunogens are able to induce antigen-specific CD4+ and CD8+ T-cell responses in the experimental animal model *in vivo*. The main aim we planned to solve in the paper was to compare influence of proteasomal and lysosomal targeting of artificial polyepitope construct on its immunogenicity and to show which of three suggested strategies (*polyE*, or ER-signal_*polyE*_LAMP-1, or Ub_*polyE*) was the most promising. Since systems of lysosome-dependent and ubiquitin-proteasome-dependent processing of antigens are rather conservative in humans and mice [55–59], we used experimental model of BALB/c mice. Taking into account that T-cell response is restricted by MHC class I molecules, we included into polyepitope construct eight marker peptides presented in a complex with both human (HLA A*02) and inbred mice BALB/c (H-2^d^) MHC class I molecules. The sequence of polyepitope fragment containing marker epitopes was also optimized for proteasomal/immunoproteasomal processing using PolyCTLDesigner software. As it’s described here, these epitopes were used only for studying immunogenicity that allow us discrimination of the effect of Ub- and (ER+LAMP1)-dependent processing of target immunogen.

After the detection of target gene expression *in vitro* it was essential to show the immunogenic potency of artificial constructs TCI-N, TCI-N2, and TCI-N3, designed as DNA vaccines, to elicit HIV-specific CD4+ and CD8+ T cell responses following immunization. For this aim we compared CD4+ and CD8+ T cell responses after three injections of naked DNA plasmids. Recently IL-2 and IFN-γ have been used as a key markers which do seem to correlate with viral control in HIV-infection [60,61], and associated with Th1 responses [60,61]. Production of IL-2 and IFN-γ activated cytotoxic CD8+ T cells and initiated the killing of infected cells that can help control of viremia [60]. We observed that all designed polyepitope immunogens induced significantly higher levels of IL-2 and IFN-γ produced by CD4+ and CD8+ T-lymphocytes after stimulation with HIV-1 peptides. These data suggest that obtained polyepitope immunogens TCI-N, TCI-N2, and TCI-N3 may be processed and presented to the CD4+ and CD8+ T cells *in vivo*.

At the next step we estimated the impact of additional Ub and LAMP-1+ER-signal sequences in the structure of immunogens on the magnitude of HIV-specific CD4+ and CD8+ T cell responses after series of immunization. We showed that the immunogens with ubiquitin and LAMP-1+ER-signal sequences induced higher magnitude of HIV-specific CD4+ and CD8+ T cell responses compared to those induced by the immunogen without additional signaling sequences. These data suggest potential immunologic advantages for polyepitope immunogens which contain ubiquitin and LAMP-1+ER-signal sequences. At the same time comparative study of the immunogenicity of the obtained vaccine constructs showed that polyepitope immunogen TCI-N3 with N-terminal ubiquitin generates the most significant levels of CD4+ and CD8+ T cell responses according to their production of IL-2 and IFN-γ after three immunizations in our study. Obtained data are consistent with both theoretical assumptions and other studies showing that covalent attachment of ubiquitin increase immunogenicity of DNA-vaccine [8,62].

Our results indirectly suggest that enhancement of *poly*E construct immunogenicity with attached ubiquitin and (LAMP-1+ER)-signal sequences is caused most probably by effective processing and presentation of polyE construct. Indeed, according to the literature, enhancement of processing and presentation of endogenously synthesized antigens results in increasing of generation of [MHC-peptide] complexes on the surface of antigen-presenting cells and enhancement of immunogenicity [52,63].

Interestingly that this effect was found not only in the context of CD8+ T cell stimulation, but also for CD4+ T cell responses. A possible explanation is that as in the case of cross-priming, which provides antigen delivery from the MHC-II pathway to MHC-I [19], mechanism was revealed which enables to direct a part of molecules from MHC-I to MHC-II pathways. This mechanism was described as “leakage”, release of antigen within apoptotic vesicles (immune apoptosis), and transport of cytosolic material into the MHC-II pathway via autophagy [62,64,65]. Perhaps, for this reason we observed also CD4+ T cell responses after immunization with DNA-vaccine construct encoding TCI-N3 (Ub_*polyE*) immunogen.

In conclusion, our data demonstrate that following immunization all investigated polyepitope immunogens elicited HIV-specific CD4+ and CD8+ T cell immune responses. TCI-N3 and TCI-N2 immunogens which contained ubiquitin and LAMP-1+ER-signal, respectively, showed statistically significant increase of CD4+ and CD8+-mediated HIV-specific responses in comparison with TCI-N immunogen without additional signaling sequences. Moreover, following vaccination TCI-N3 (Ub_*polyE*) polyepitope immunogen generated highest levels of IL-2 and IFN-γ expression by CD4+ and CD8+ T cell lymphocytes. Our data suggest that optimization of polyepitope HIV-1 immunogen structure may influence on magnitude of CD4+ and CD8+ HIV-specific responses. These results support the strategy of the optimal HIV-1 T cell immunogen design and may contribute to the development of effective HIV-vaccine.
